# Cadherin-23 Mediates Heterotypic Cell-Cell Adhesion between Breast Cancer Epithelial Cells and Fibroblasts

**DOI:** 10.1371/journal.pone.0033289

**Published:** 2012-03-07

**Authors:** Maria Apostolopoulou, Lee Ligon

**Affiliations:** Department of Biology and Center for Biotechnology and Interdisciplinary Studies, Rensselaer Polytechnic Institute, Troy, New York, United States of America; University Medical Center Utrecht, The Netherlands

## Abstract

In the early stages of breast cancer metastasis, epithelial cells penetrate the basement membrane and invade the surrounding stroma, where they encounter fibroblasts. Paracrine signaling between fibroblasts and epithelial tumor cells contributes to the metastatic cascade, but little is known about the role of adhesive contacts between these two cell types in metastasis. Here we show that MCF-7 breast cancer epithelial cells and normal breast fibroblasts form heterotypic adhesions when grown together in co-culture, as evidenced by adhesion assays. PCR and immunoblotting show that both cell types express multiple members of the cadherin superfamily, including the atypical cadherin, cadherin-23, when grown in isolation and in co-culture. Immunocytochemistry experiments show that cadherin-23 localizes to homotypic adhesions between MCF-7 cells and also to heterotypic adhesions between the epithelial cells and fibroblasts, and antibody inhibition and RNAi experiments show that cadherin-23 plays a role in mediating these adhesive interactions. Finally, we show that cadherin-23 is upregulated in breast cancer tissue samples, and we hypothesize that heterotypic adhesions mediated by this atypical cadherin may play a role in the early stages of metastasis.

## Introduction

Fibroblasts are the most abundant cell type found in the stroma surrounding glandular epithelial tissues. Ample evidence indicates that fibroblasts can promote epithelial tumor progression via paracrine signaling, but direct contact with invading tumor cells may affect tumorigenesis and metastasis as well [1]. Recent evidence has shown that fibroblasts and epithelial cells can form heterotypic cell-cell adhesions *in vitro*, and that cadherin adhesion molecules are recruited to these heterotypic adhesions [2].

Metastatic tumor cells often show changes in cadherin expression [3]. Many tumor cells undergo a “cadherin switch” in which E-cadherin expression decreases and N-cadherin expression increases, although changes in other cadherins may also be observed. These changes in cadherin expression are associated with the decreased adhesion and increased motility characteristic of metastatic disease [4]–[6].

Cadherin-23 is an atypical cadherin implicated in several deafness syndromes due to its role as a component of the tip link complex between stereocilia in cochlear hair cells [7], [8]. It has up to 27 extracellular cadherin repeats (ECs) (Fig. S1A) [9] and a unique cytoplasmic domain that interacts indirectly with the actin cytoskeleton [10]. Although the N-terminal EC (EC1) differs sufficiently from that of classical cadherins to warrant the classification of cadherin-23 as a cadherin-related protein [7], [11], biochemical and structural data suggest that cadherin-23 can form Ca^2+^-dependent homophilic adhesions through its EC1 [12]. Several isoforms, differing in size, EC number, tissue and developmental expression, have been described, although little is known about the functional significance of these isoforms [13], [14].

Here we sought to determine if breast cancer epithelial cells can make heterotypic cell-cell adhesions with normal fibroblasts and to test whether members of the cadherin superfamily mediate these interactions. We found that MCF-7 breast cancer cells and normal breast fibroblasts (NBFs) form heterotypic adhesions in a co-culture model of the tumor microenvironment, and we found that an atypical member of the cadherin superfamily, cadherin-23, localizes to sites of heterotypic contact. We also found that disruption of cadherin-23 expression or function decreases both homotypic adhesion between epithelial cells and heterotypic adhesion between epithelial cells and fibroblasts. Finally, we show that cadherin-23 is up-regulated in breast cancer tissue versus normal tissue and we propose that cadherin-23-mediated heterotypic adhesion between invading tumor cells and stromal fibroblasts may play a role in the metastatic cascade.

## Results and Discussion

### MCF-7s and NBFs form heterotypic adhesions *in vitro*

**Figure 1:**
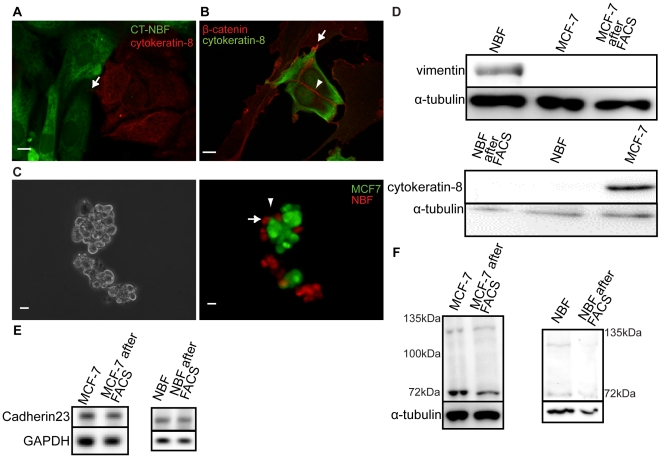
MCF-7s and NBFs form heterotypic adhesions and express cadherin-23 *in vitro*. A) NBFs were pre-labeled with CellTracker™ Green and co-cultured with MCF-7s for 3 days. Cells were then fixed and labeled with an antibody against cytokeratin-8 (red). Arrow shows a site of heterotypic contact between the two cell types. B) Three-day co-cultures labeled with antibodies against β-catenin (red) and cytokeratin-8 (green). Arrow – heterotypic contact; Arrowhead – MCF-7 homotypic contact. Scale = 10 µm. C) MCF-7s labeled with CellTracker Green™ (arrowhead) were co-cultured with NBFs labeled with CellTracker Red™ (arrow). Phase contrast (left) and fluorescence image (right) of a typical heterotypic cell aggregate are shown. Scale = 20 µm. D) Immunoblots show no vimentin expression in MCF-7 fraction and no cytokeratin-8 in NBF fraction after FACS separation of co-cultures. PCR (E) and immunoblots (F) showing cadherin-23 expression in MCF-7s and NBFs before and after co-culture. α-tubulin and GAPDH were used as controls.

To determine if MCF-7 cells and NBFs can form heterotypic cell-cell adhesions, we developed a co-culture system in which we grew cells together for up to three days. To distinguish between the two cell types after co-culture, we either pre-labeled one or both cell types with CellTracker™ dyes before co-culture (Fig. 1A, C), and/or fixed the cultures and performed immunocytochemistry with antibodies to cell-type-specific intermediate filaments (cytokeratin-8 for MCF-7s (Fig. 1A, B) and vimentin for NBFs (data not shown)) [15], [16]. We also used an antibody to β-catenin to identify sites of possible cell-cell adhesion, as β-catenin is a cytoplasmic partner of many cadherin family members (Fig. 1B). We found that when MCF-7s and NBFs were in close proximity, they extended cytoplasmic processes to make contact with one another (Fig. 1A, B, arrows). We scored these contacts for β-catenin recruitment and found it at 82.1% of heterotypic contacts (n = 451, Fig. 1B, arrow). We also noted that both cell types retained their distinct morphologies in co-culture (i.e. fibroblasts are long, spindle-shaped cells, while MCF-7s are more round and compact), and these characteristic phenotypes were used to aid in distinguishing cell types in some subsequent experiments.

We then performed adhesion assays to test the strength of these interactions. Each cell type was pre-labeled with a CellTracker™ dye (Green or Red), then equal numbers of epithelial cells and fibroblasts were combined in agarose-coated wells (to prevent cell-surface adhesion). After one hour of rotating incubation, cell aggregates were counted (n = 515) and scored for the presence of each cell type. 47.2% of the aggregates contained both cell types (Fig. 1C), suggesting that MCF-7s and NBFs can form heterotypic adhesions in a simplified system in which the role of cell:ECM adhesion is minimized.

### Both MCF-7s and NBFs express cadherin-23

We hypothesized that the heterotypic adhesion we observed may be mediated by cadherins, so we screened cells cultured alone as well as co-cultured cells by PCR for expression of cadherin family members (Table S1). As expected, MCF-7s express epithelial cadherins such as E- and P-cadherin and NBFs express fibroblast cadherins such as N- and OB-cadherin, and all these cadherins were also expressed in co-cultures (Table S1). Surprisingly, the atypical cadherin-23 was also expressed by both cell types alone and in co-cultures (Table S1, Fig. S1B).

As the expression of cadherin-23 has only previously been reported in the neurosensory epithelium, we confirmed its expression with two independent PCR primer sets and then cloned and sequenced the PCR products of the MCF-7 monocultures and the 3-day co-cultures, and found that they were >99% identical to cadherin-23 (via BLAST comparison). We noted that the expression of cadherin-23 was typically lower in fibroblasts than in the MCF-7 cells, which we confirmed with qPCR (Fig. S1D). We then confirmed cadherin-23 protein expression with immunoblotting (Fig. S1C), and found that two major isoforms (∼70 and ∼130 kD) were expressed both by mono- and co-cultures. Neither cell type showed any detectable expression of the longest predicted isoform, and the MCF-7s consistently showed higher cadherin-23 protein expression than NBFs (Fig. S1C). Together, these data suggest that cadherin-23 is expressed by both cell types alone and in co-cultures, but that the protein is expressed at higher levels in epithelial cells than in fibroblasts.

To verify that cadherin-23 is expressed by each cell type in co-culture, we separated co-cultured cells using FACS after first labeling one cell type with CellTracker™. Efficient separation was confirmed using cytokeratin-8 and vimentin immunoblotting (Fig. 1D). We then probed the separated cell populations for cadherin-23 expression and compared it to the levels found in mono-cultures and found that for each cell type, cadherin-23 expression levels were similar in mono- and co-cultures (Fig. 1E, F).

### Cadherin-23 localizes to homotypic and heterotypic contact sites

**Figure 2:**
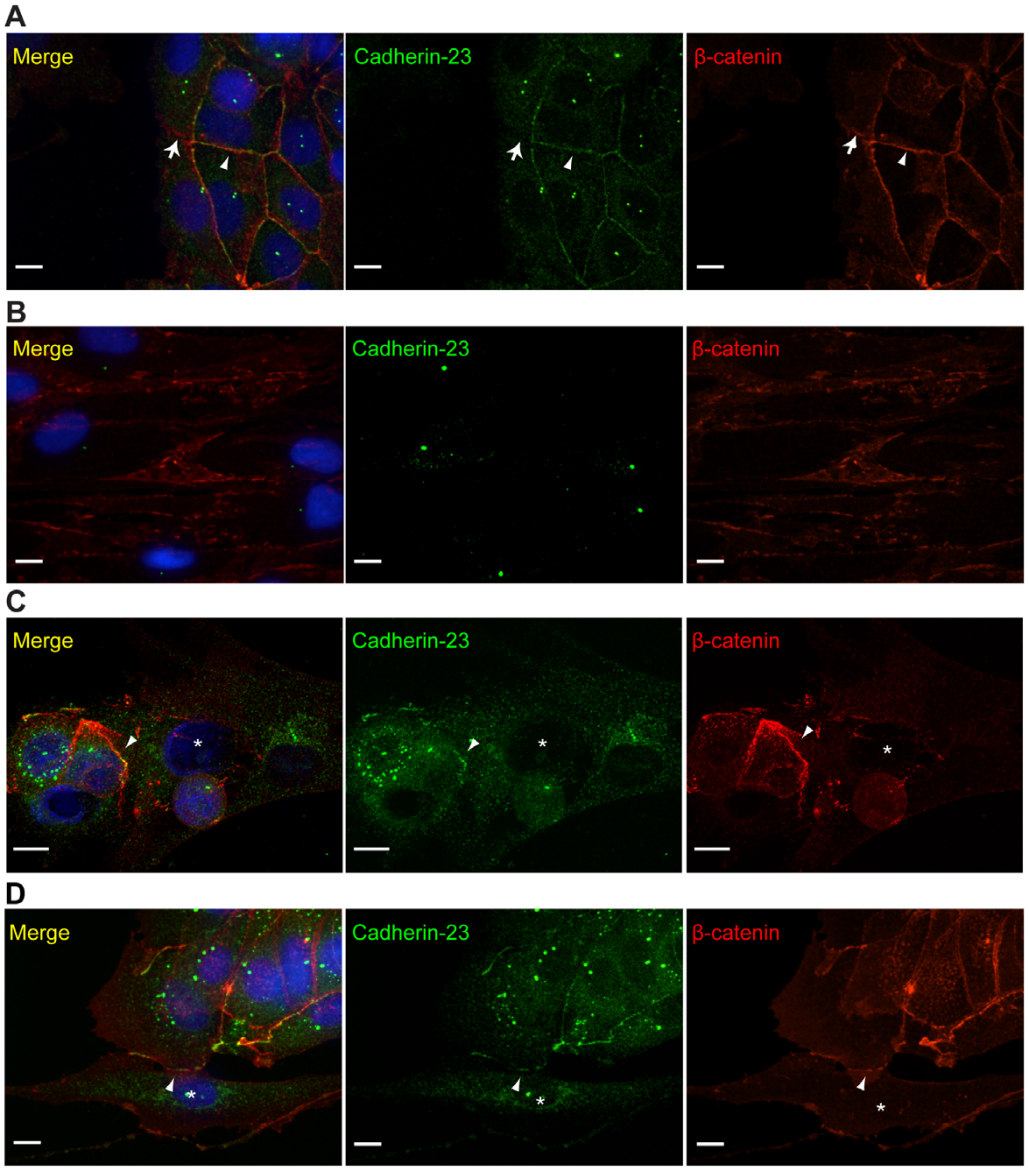
Cadherin-23 localizes to homotypic and heterotypic contacts. Cadherin-23 localizes at 75% of homotypic contacts between MCF-7s (arrowhead, A) but not NBFs (B). Arrow in (A) points to a contact between MCF-7s without cadherin-23 recruitment. Cadherin-23 localizes at 23% of heterotypic adhesions in two-hour (C) and 45% of three-day (D) co-cultures (arrowhead). Asterisks in (C–D) mark fibroblasts. Fig. 2C shows MCF-7 cells and NBFs co-cultured for two hours, where a group of spherical MCF-7 cells is on top of a flat and extended fibroblast (asterisk). One of the MCF-7 cells has formed a possible adhesion site with the fibroblast, as evidenced by β-catenin labeling, and cadherin-23 has already been recruited at that site (arrowhead). Fig. 2D shows a group of compact MCF-7 cells, where one of them has formed an adhesion site with an extended fibroblast (asterisk), as marked by β-catenin localization, where cadherin-23 has been recruited (arrowhead). All images are maximum projections of confocal z-stacks. Scale = 10 µm.

To determine if the cadherins expressed were localized to homotypic and/or heterotypic contact sites, we performed immunocytochemistry on mono- and co-cultures with antibodies to each cadherin in combination with an antibody to β-catenin which marked possible cell-cell adhesion sites (Fig. S2, Fig. 2). As expected, E- and N-cadherin were robustly localized at homotypic adhesions between MCF-7s and NBFs, respectively (Fig. S2A, B, arrows). Both cadherins also localized to heterotypic contacts, but neither labeled the entire contact site (Fig. S2A, B, arrowheads). P- and OB-cadherin localized to homotypic adhesions between MCF-7s and NBFs, respectively, but not to heterotypic contacts (Fig. S2C, D).

Cadherin-23 was not localized at homotypic adhesions between NBFs, but was found at 75.4% of homotypic adhesions between MCF-7s (n = 61 cell pairs) (Fig. 2A, B). It was typically found at contacts between cells in the interior of the epithelial cell island, but was often absent from contacts at the island boundary (Fig. 2A, arrow). Cadherin-23 localized to 23% of heterotypic contacts between MCF-7s and NBFs two hours after co-culturing (Fig. 2C, arrowhead), and by 3 days was found at 45% of heterotypic cell pairs (Fig. 2D, arrowhead) (n = 40 and n = 100 cell pairs, respectively). These results suggest that E- and N-cadherin, as well as cadherin-23 are localized at heterotypic contacts, where they may play a role in heterotypic adhesion.

In NBFs, antibodies to cadherin-23 also showed a prominent localization to centrosomes (Fig. 2B), where they co-localized with an antibody to γ-tubulin (data not shown). Cadherin-23 has previously been shown to localize to centrosomes in the early stages of inner ear hair cell development [13], but the physiological significance of this localization is unclear.

### Cadherin-23 participates in MCF-7 homotypic and heterotypic adhesion

To determine if cadherin-23 plays a role in adhesion, we performed adhesion assays after disruption of cadherin-23 with inhibitory antibodies or after knockdown of the protein by RNAi.

To disrupt cadherin-23 function, we used an antibody to cadherin-23 EC1, which is thought to be the primary adhesion domain [11], [12]. As a positive control for MCF-7 homotypic adhesion, we used an antibody to E-cadherin EC1, which interferes with E-cadherin mediated adhesion [17]. As a negative control for MCF-7 homotypic adhesion, we used an antibody to OB-cadherin which disrupts adhesion in OB-cadherin expressing cells [18], but would not be expected to have an effect in MCF-7s as they do not express the protein. As a negative control for heterotypic adhesion, we used a no-antibody treatment. We performed the adhesion assays as described above and measured the increase in cell aggregates between plating and 2 hours of rotating incubation.

**Figure 3:**
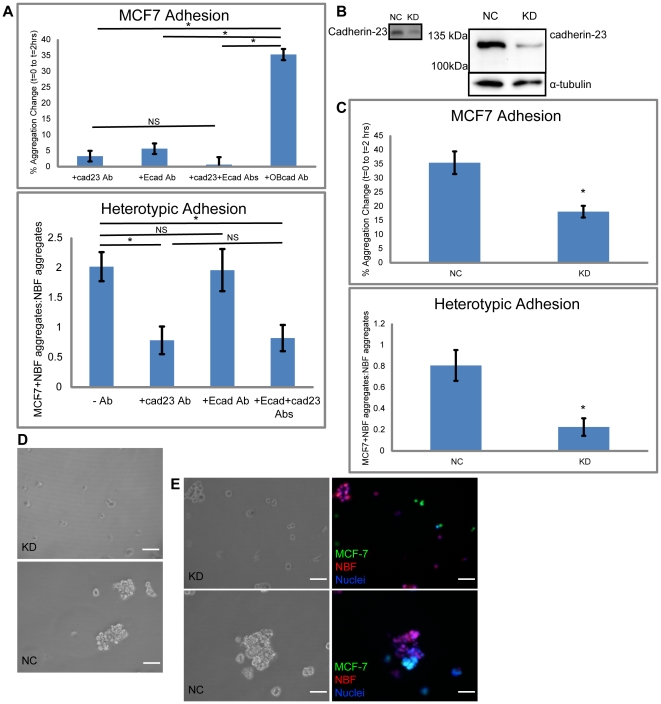
Cadherin-23 plays a role in homotypic and heterotypic adhesion. A) Adhesion assays using inhibitory antibodies. Top panel: the percent of total MCF-7 cell aggregates comprised of 3 or more cells was measured at t = 0 and at t = 2 hrs and the change in this percentage was calculated. Cadherin-23 and E-cadherin antibodies, individually and in conjunction, showed a statistically significant reduction of adhesion when compared to the effect of an antibody that blocks OB-cadherin-mediated adhesion. (n = 3 independent experiments; one tail unpaired t-test; asterisks mark statistically significant differences). Bottom panel: adhesion assays on 1∶1 mixture of MCF-7:NBF after 2 hrs. The antibody against cadherin-23, both alone and in conjunction with the E-cadherin antibody, significantly reduced heterotypic adhesion. (n = 6 independent experiments; one tail unpaired t-test; asterisks mark statistically significant differences; NS = not significant). B) RNAi knock down of cadherin-23 in MCF-7 cells, as evidenced by both RT-PCR (left panel) and immunoblotting (right panel). In the left panel, 25 ng of mRNA were used for each PCR reaction, and the entire product was run on the agarose gel. NC: non-coding RNAi sequence; KD: cadherin-23 specific RNAi sequence. C) Adhesion assays, as in panel 3A, but after RNAi knock down of cadherin-23 in MCF-7 cells. Top panel: RNAi treatment significantly reduced the ability of MCF-7 cells to participate in homotypic adhesion. (n = 3; one tail unpaired t-test; asterisks mark statistically significant differences). Bottom panel: RNAi treatment significantly reduced the number of heterotypic aggregates after 2 hrs. (n = 3; one tail unpaired t-test; asterisks mark statistically significant differences). D) Representative image of MCF-7 cell aggregates after treatment with RNAi. Scale = 100 µm. E) Representative image of MCF-7:NBF cell aggregates after treatment with RNAi. shRNA plasmids contained a gene to a green fluorescent protein, while NBFs were labeled with CellTracker™ Red. Hoescht dye was used to stain the nuclei.

Both the E-cadherin and the cadherin-23 antibodies individually and in combination significantly reduced MCF-7 homotypic aggregation after two hours in comparison to the OB-cadherin antibody (Fig. 3A, top panel) or no antibody (data not shown), suggesting that both proteins may play a role in epithelial cell-cell adhesion. The cadherin-23 antibody shows no reactivity to E-cadherin by immunocytochemistry or immunoblotting (data not shown), and the effects of the E-cadherin and cadherin-23 antibodies in combination is greater than that of either alone, suggesting that the effect of the cadherin-23 antibody is not merely due to non-specific interaction with E-cadherin. Neither the E-cadherin antibody nor the cadherin-23 antibody affected NBF homotypic cell aggregation (data not shown), presumably because neither protein is localized at NBF homotypic contact sites (Fig. 2B and data not shown).

To test the role of cadherin-23 in heterotypic adhesion, we pre-labeled the two cell populations with different CellTracker™ dyes (Green and Red), performed adhesion assays as above, and then compared the number of MCF-7/NBF heterotypic aggregates to NBF homotypic aggregates. We used the number of NBF homotypic aggregates as a standard because they were not affected by either antibody treatment (above). Cadherin-23-antibody incubation inhibited heterotypic adhesion while the E-cadherin antibody alone did not affect heterotypic adhesion (Fig. 3A, bottom panel). Both antibodies combined did not show any additional effect.

To verify the results of the antibody inhibition experiments, we knocked down cadherin-23 expression in MCF-7 cells using RNAi, as evidenced by RT-PCR and immunoblotting (Fig. 3B) and then performed adhesion assays as described above. As the shRNA plasmids also coded for a green fluorescent protein, we used CellTracker Red™ to label the NBFs. RNAi knockdown of cadherin-23 significantly affected the ability of MCF-7 cells to participate in both homotypic and heterotypic adhesion, consistent with the results obtained from the antibody inhibition experiments (Fig. 3C). Typical homotypic and heterotypic cell aggregates shown in Fig. 3D–E. Both types of cell aggregates from cells expressing cadherin-23 (NC) are consistently much larger than those in which cadherin-23 expression is decreased (KD). Together, these data suggest that both E-cadherin and cadherin-23 play a role in MCF-7 homotypic adhesion, but that only cadherin-23 and not E-cadherin has a role in heterotypic adhesion.

In the inner ear stereocilia, protocadherin-15 is thought to be the binding partner for cadherin-23 [19]. However, we failed to detect protocadherin-15 expression in MCF-7s, NBFs or co-cultures (data not shown). Cadherin-cadherin binding is highly class specific because of the strand-swapping between cadherin domains. It has been suggested that cadherin-23 can participate in homophilic adhesion, but the mechanism is likely to differ from that of classical cadherins because of structural differences between the cadherin domains [11], [12]. Therefore, although E- and N-cadherin partially co-localize with cadherin-23 at heterotypic adhesions, it is unlikely that they can serve as heterophilic binding partners with cadherin-23. Our evidence that the E-cadherin antibody did not affect heterotypic adhesion supports this hypothesis, and therefore, we think it most likely that heterotypic adhesion between tumor cells and fibroblasts is mediated by cadherin-23 homophilic adhesion.

### Cadherin-23 expression is upregulated in breast carcinoma tissues

**Figure 4:**
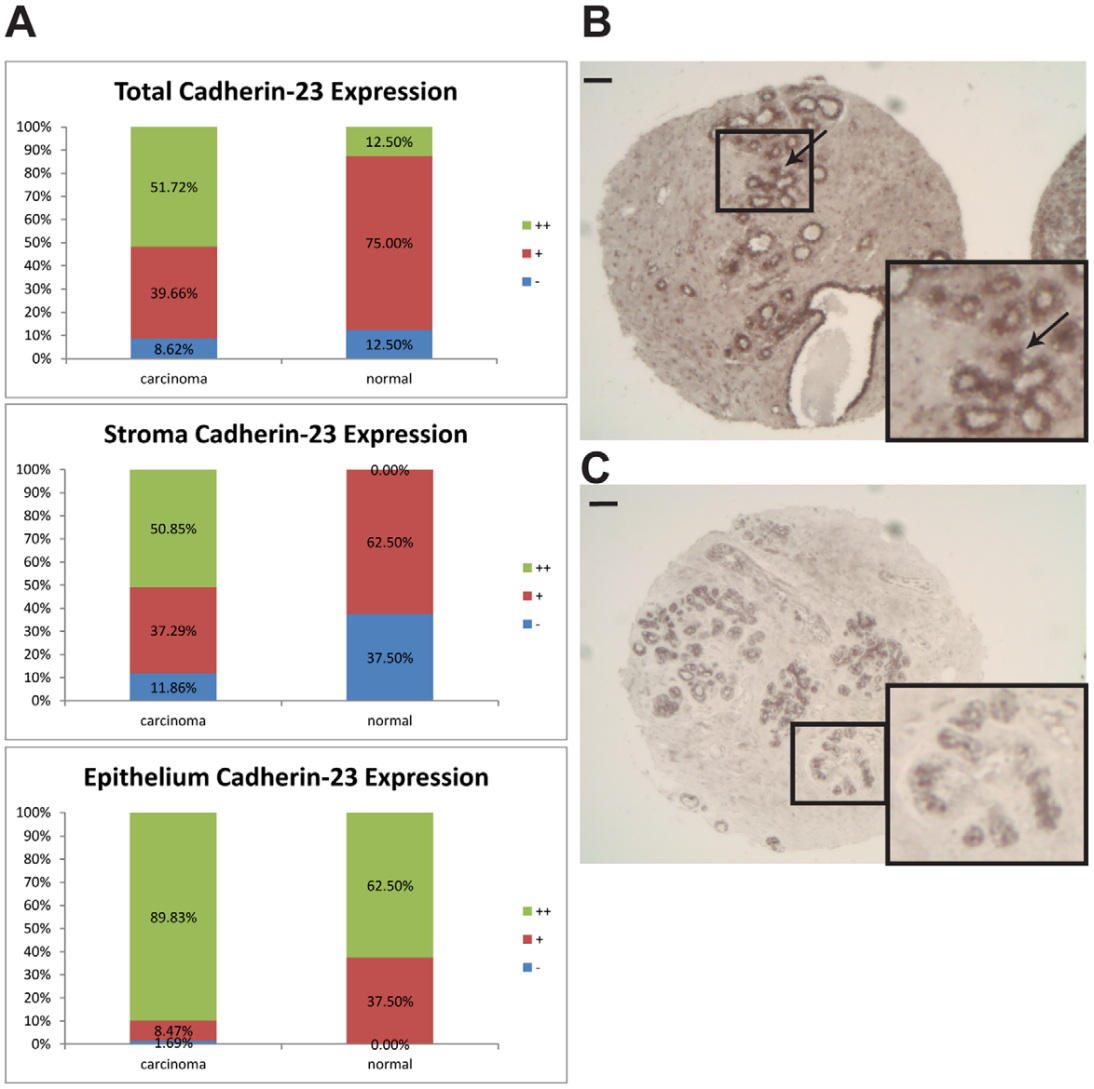
Cadherin-23 is upregulated in breast cancer. Samples from breast cancer (n = 58, including both *in situ* and invasive carcinomas) and healthy breast tissue (n = 8) were labeled with cadherin-23 antibodies and blindly scored for overall (top panel), stromal (middle panel) and epithelial (bottom panel) cadherin-23 labeling (‘−’: no expression; ‘+’: low expression; ‘++’: high expression) (A). In all cases carcinoma samples showed significantly higher expression (Wilcoxon Rank-Sum test, p = 0.01). Representative carcinoma (*in situ*) (B) (scored as ‘++’) and normal breast (C) tissue (scored as ‘−’) labeled with cadherin-23 antibodies. Arrow in (B) points to a duct with intense cadherin-23 labeling that is budding into the stroma. Insets in (B–C) show a higher magnification of the enclosed area. Scale = 0.1 mm.

To determine whether cadherin-23 may play a role in human breast cancer, we labeled a breast cancer tissue microarray with cadherin-23 antibodies. Samples were blindly scored for overall level of cadherin-23 expression and tumor samples showed significantly higher cadherin-23 expression than healthy tissue (Fig. 4A, top panel). Increased cadherin-23 expression was seen in both epithelial and stromal cells in cancer samples (Fig. 4A, middle and bottom panels and B–C). In both tumor and healthy samples, cadherin-23 expression was higher in luminal epithelia than in stroma, consistent with the higher cadherin-23 expression we observed in MCF-7s compared to NBFs (Fig. 4B–C, S1B–C). Interestingly, we often observed strong stromal tissue labeling adjacent to intensely stained luminal tissue in many disease samples, but not in healthy breast tissue (Fig. 4B–C, arrow). Together these data suggest that cadherin-23 is expressed in human ductal epithelial cells, is up-regulated in human breast cancer and further that the increased stromal labeling may serve to mediate heterotypic adhesion between invading tumor cells and stromal fibroblasts.

Cadherin-23 may be involved in signaling as well. Cadherin-23 belongs to the cadherin-related-2 subfamily [7] and it has a cytoplasmic PDZ binding motif [20], [21]. Another member of the same subfamily with a PDZ-binding motif, protocadherin-24, has been shown to play a role in regulating contact inhibition [22]. Cadherin-23 is also 80% similar to Fat1, a cadherin with roles in tumor suppression and planar cell polarity [23], [24]. We observed that in confluent MCF-7s, cadherin-23 is localized to all cell contacts, but in sub-confluent cultures, peripheral cell adhesions usually lack cadherin-23 (Fig. 2A). These results suggest that cadherin-23 may play a role in contact inhibition or planar cell polarity, but further experiments will be necessary to elucidate this role.

## Materials and Methods

### Cell Culture

MCF-7s and NBFs (CCD-1065SK) from ATCC (Manassas, VA, USA) were grown in MEM/EBSS (Hyclone, Logan, UT, USA) containing 2 mM L-glutamine (Hyclone), 10% FBS (Atlanta Biologicals, Laurensville, GA, USA), 1 mM Na-Pyruvate (Sigma, St. Louis, MO, USA) and 100 µg/ml Penicillin/Streptomycin (Hyclone). MCF-7 and co-culture media also contained insulin (0.01 and 0.005 mg/ml, respectively) (GIBCO, Carlsbad, CA, USA). We verified that each cell type grew equally well in co-culture media with growth curves. The fibroblast cell line used in these studies was derived from the mammary skin of a breast cancer patient after surgery. As such, these fibroblasts are not cancer-associated or activated, as was evidenced by the absence of α-smooth-muscle-actin mRNA expression (data not shown). Although these are skin fibroblasts, they are thought to model stromal fibroblasts [25].

### Immunocytochemistry

Cells were grown on glass coverslips, fixed in anhydrous methanol containing 1 mM EGTA at −20°C and processed with β-catenin (Zymed, San Fransisco, CA, USA), cytokeratin-8 (Sigma), vimentin (Sigma), N-cadherin (BD, Franklin Lakes, NJ, USA), E-cadherin (Chemicon, Billerica, MA, USA), P-cadherin (R&D Systems, Minneapolis, MN, USA), OB-cadherin (Chemicon) or cadherin-23 antibodies (Abnova, Cat. # H00064072-A01, Taipei City, Taiwan), followed by Alexa Fluor 488 and 594 secondary antibodies (Molecular Probes, Carlsbad, CA, USA) and DAPI (Invitrogen, Carlsbad, CA, USA) to counterstain the nuclei. Coverslips were mounted with ProLong (Molecular Probes). Images were acquired at room temperature using Volocity (Improvision, Waltham, MA, USA) on a Leica microscope with a Yokogawa spinning disk, a Plan-Apo 63X 1.3 N.A. glycerol objective and an ORCA-ER camera (Hamamatsu) or using Zeiss AIM (Jena, Germany) on a Zeiss LSM 510 confocal microscope with a Plan-Apo 63X 1.3 N.A. oil objective. Color balance was adjusted using Adobe Photoshop CS2 (San Jose, CA, USA). The percentage of adhesion sites that recruited cadherin-23 was calculated by scoring the possible adhesion sites (which showed β-catenin localization) for cadherin-23 labeling. For example, in Fig. 2A, the arrowhead points to an adhesion site that recruits cadherin-23, while the arrow points to an adhesion site that shows β-catenin localization, but not that of cadherin-23.

### Adhesion Assays

MCF-7s or NBFs (labeled with CellTracker™ Green or Red (Invitrogen)) were suspended using CellStripper™ (Cellgro, Manassas, VA, USA). Only cultures with <20% aggregates with 3 or more cells after suspension were used in adhesion assays. 80,000–100,000 cells (of a single type or a 1∶1 mixture) were suspended in Ca^2+^-containing HBSS (Hyclone) with 0.02 U/µl DNAse I (NEB, Ipswich, MA, USA) and Hoescht dye (Sigma), and added to 1%-agarose-pre-coated tissue culture plates (d = 2.5 cm), which were placed on a rotating shaker (80 rpm) at 37°C. For MCF-7 adhesion assays, >100 cell aggregates were randomly chosen and scored for having 3-and-more or <3 cells. The 3-and-more cells aggregate percentage change was calculated between plating and after two hours. For heterotypic adhesion assays, >50 cell aggregates (2 or more cells) were randomly chosen and scored for cell composition (MCF-7 only, NBF only or MCF7+NBF) after 2 hrs of rotating incubation. The number of MCF7+NBF aggregates was compared to NBF-only cell aggregates (MCF7+NBF/NBF) (y axis at bottom panels of Fig. 3A, C). Antibody-inhibition experiments were performed with 1∶250 cadherin-23 antibody (against the EC1 domain, Abnova, Cat. # H00064072-A01), 1∶100 E-cadherin antibody (67A4, Millipore, Billerica, MA, USA), and 1∶250 OB-cadherin antibody (R&D Systems, Cat. # AF1790). The cadherin-23 antibody used did not recognize a band of the same size as E-cadherin (120 kDa) during immunoblotting experiments.

### RT-PCR

Confluent monocultures of MCF-7s, NBFs, or 1-hour or 3-day co-cultures were trypsinized and ∼20×10^4^ cells were used for mRNA isolation (RNEasy, Qiagen, Valencia, CA, USA) for RT-PCR (Cells-to-Signal, Ambion, Austin, TX, USA). PCR primers and conditions for E- and N-cadherin were as in [26]. Other primers were P-cadherin: 5′-TCCTCCGCCTCCGACCAAGAC-3′ and 5′-GGCCCAGGCCCAGGACACA-3′; R-cadherin: 5′-CAAAGTTGGGGCAGATGGGACAGT-3′ and 5′-GCGTGGGCTCGGAGGTGGTAAGA-3′; VE-cadherin: 5′-CCCGGAGTTTGCCAAGCCCTACCA-3′ and 5′-ATCTCGCCGCCGCCCTCCTC-3′; K-cadherin: 5′-GACAACACGGCGGGAATCT-3′ and 5′-AAGGAGTCGTATGGCGGGGCAGTG-3′; cadherin 7: 5′-CCAGCCGGGGCAGGTTATCCAGA-3′ and 5′-CTCCCCCGCCCTCGTCATCGTATC-3′; cadherin 8: 5′-AAAACCCGGCCAAGTCATTCAA-3′ and 5′-CTCCCCTCCTCCTTCATCATCGTA-3′; T1-cadherin: 5′-GTGTTGTTTGCTGCATTGAAGAGG-3′ and 5′-TTGAAACGAGGCCCCCAGTC-3′; T2-cadherin: 5′-ACGCGTGGCTGTTTTTGTGAGA-3′ and 5′-GAGGCCGGCAGGGAGGAG-3′; OB-cadherin: 5′-AGCGGCCCTGTGACATTCCTTC-3′ and 5′-CCTGCCCCTCCTTGCCCTTCTCAT-3′; N-cadherin 2: 5′-TGGCGCTGTCACTGCTCAA-3′ and 5′-CCTGCGGCTGTATCCATTTCTT-3′; H-cadherin: 5′-GCGGAAGATATGGCAGAACTCGTG-3′ and 5′-CATTATCGGTGGCTGGGTCATCTG-3′; M-cadherin: 5′-CCCTGGACATCGCCGACTTCATCA-3′ and 5′-GGCCGCCCCATCCACACTCAGGT-3′; KSP-cadherin: 5′-CCTCGGCTGGGGGCTCTGG-3′ and 5′-CGGGGGCATCTGCATCCTCTG-3′; LI-cadherin: 5′-CAAGCGAAAGTCAGTGAGGATGTA-3′ and 5′-ACTGCCGAGGGAAAATGTAAAATG-3′; cadherin18: 5′-ACGCCTGCCTGTAAATCCAAACT-3′ and 5′-TCTCCGCCTCCTTCATCATCATAG-3′; cadherin 19: 5′-GAACGCAAGACTCGCAAAACC-3′ and 5′-CCATAGAGCCAGGGCACAAAAC-3′; cadherin 20: 5′-GCGGCACCGGAAACAACCATACAT-3′ and 5′-GCCTGCTCACCCCCATTCTCACTC-3′; cadherin 22: 5′-AGCAGCAGCGGCGGCGATGTGTT-3′ and 5′-CGCGGTCCAGCCCCTTGCCAGTC-3′; cadherin 23 (EC1): 5′-CCGGCTGCCCTTCTTCACCAACCA-3′ and 5′-ACGATACCGCGGGCGCTGTCAATG-3′; cadherin 23 (EC1-2): 5′-CCGGCTGCCCTTCTTCACCAACCA-3′ and 5′-ATGGTGTAGCCAATGCCCCG-3′; cadherin 24: 5′-CCGCGGCTGACCTGGACT-3′ and 5′-GGGCCCGGATGACCTGAA-3′. Products were run on a 1%-agarose (Fisher, Waltham, MA, USA) gel with 0.0001% ethidium-bromide (Fisher). Bands of the expected size were registered as ‘+’ on Table S1, otherwise as ‘−’. PCR conditions were 35 cycles of: 94°C for 1.5 min, 45°C for 2 min and 72° for 3 min. PCR was performed on a BioRad MyCycler (Hercules, CA, USA). Products were extracted (QIAquick, Qiagen), reamplified with Phusion Taq polymerase (Invitrogen), cloned using BLUNT Zero (Invitrogen), and sent for sequencing at Eurofins MWG Operon (Huntsville, AL, USA).

### Immunoblotting

Confluent MCF-7 and NBF mono-cultures and 1-hour and 3-day co-cultures were lysed in RIPA buffer (50 mM Tris, 150 mM NaCl, 6 mM Na-deoxycholate, 1% Triton-X 100, 0.1% SDS, 2 mM EDTA, pH 8.0) containing Protease Inhibitor Cocktail III (RPI Corp., Mt. Prospect, IL, USA). Lysates were centrifuged at 13,500 rpm at 4°C for 5 min and the supernatant was mixed with sample buffer (0.125% bromophenol-blue in Tris-Cl, pH 6.8, 25% glycerol, 10% SDS, 40 mM DTT) in a 1∶1 ratio at 100°C for 20 mins. Proteins were separated using SDS-PAGE and transferred onto an Immobilon-P membrane (Millipore), blocked with 5% milk and labeled with the aforementioned antibodies (along with an antibody against the cytoplasmic domain of cadherin-23 from Sigma, Cat. # SAB104883), followed by HRP-conjugated antibodies (Jackson ImmunoResearch, West Grove, PA, USA), and visualized with SuperSignal West Femto (Thermo Scientific, Waltham, MA, USA) and a ChemiImager 4400 (Alpha Innotech, Randburg, South Africa). Densitometric analysis was performed using ImageJ [27].

### FACS

MCF-7s were labeled with CellTracker™ Green (Invitrogen), co-cultured with NBFs for 1 day, trypsinized and sorted with a BD Biosciences FACSAria I. RT-PCR and immunoblotting was performed as described above.

### Immunohistochemistry

Immunohistochemistry was performed on a breast cancer tissue microarray (Imgenex, San Diego, CA, USA; Cat. No IMA-100) with two cadherin-23 antibodies in conjunction (against the EC1: Abnova, Cat. # H00064072-A01; against the cytoplasmic domain: Sigma, Cat. # SAB104883) followed by Vectastain Elite ABC and DAB Substrate (Vector Laboratories, Burlingame, CA, USA). Antigen was retrieved in a 95°C water bath in 0.01 M sodium-citrate (pH 6.0) for 10 min and endogenous peroxidase activity was quenched with 3% hydrogen-peroxide for 6 min. Images were acquired at room temperature with Spot on a Zeiss SteREO microscope with a Plan-Apo 1.5× objective and a 1.3 Mp color Diagnostic Instruments camera. ImageJ [27] was used to acquire mean intensity values for each tissue sample, as well as for stromal and epithelial regions of the samples. Tissue samples that were incomplete were not included in the analysis. Tissue samples with mean intensity 0 were given the score ‘−’. Tissue samples with mean intensity above 0 but below 100 were given the score ‘+’. Tissue samples with mean intensity above 100 were given the score ‘++’.

### RNAi

shRNA plasmids specific for cadherin-23 and non-coding were ordered from SA Biosciences (SureSilencing shRNA Plasmids, Frederick, MD, USA). 4×10^6^ MCF-7 cells were transfected with 5 µg DNA using the Amaxa Cell Line Nucleofector Kit V (Lonza, Cologne, Germany) in an Amaxa Nucleofector 2 (Lonza). Cells were harvested for further experiments 24 hrs after nucleofection. RT-PCR, immunoblotting and adhesion assays were performed as described above.

### qPCR

Confluent monocultures of MCF-7s and NBFs were trypsinized and ∼20×10^4^ cells were used for mRNA isolation (RNEasy with RNAse-Free DNase set, Qiagen). qPCR was performed using the QuantiFast SYBR Green RT-PCR kit (Qiagen) on a LightCycler 480 (Roche, Indianapolis, IN). Primers used were GAPDH (reference gene): 5′-TCTTCCAGGAGCGAGATC-3′ and 5′-AGCCCCAGCCTTCTCC-3′; cadherin-23: 5′-CCGGCTGCCCTTCTTCACCAACCA-3′ and 5′-GGCCTCCTCCCCAGACACGCC-3′. PCR conditions were 45 cycles of: 95°C for 10 sec, 50°C (GAPDH) or 52°C (cadherin-23) for 25 sec and 72°C for 7 sec (GAPDH) or 9 sec (cadherin-23). Fold change was calculated according to [28].

## Supporting Information

Figure S1 **Cadherin-23 is expressed in both MCF-7 cells and NBF cells.** A) Domain structure of cadherin-23. B) PCR comparing the expression of cadherin-23 and GAPDH (as a control) in MCF-7 cells, NBF cells and cells co-cultured for 1 hour or 3 days. C) Immunoblot showing protein expression of cadherin-23 and α-tubulin (as a control) in MCF-7 cells, NBF cells and cells co-cultured for 1 hour or 3 days. D) qPCR comparing the expression of cadherin-23 in MCF-7 and NBF mono-cultures. Fold change of cadherin-23 for NBF mono-cultures was compared to that of MCF-7 mono-cultures, which was set as 1. Error bars represent standard error from three independent experiments. Asterisk denotes statistically significant difference (student's t-test, p<0.01).
(TIF)

Figure S2 **Cadherin localization at homotypic and heterotypic cell-cell contacts.** Three-day co-cultures labeled with antibodies to the noted cadherins (green) and β-catenin (red). Nuclei were labeled with DAPI (blue). A) E-cadherin is localized at homotypic adhesion sites (arrow) between MCF-7 cells, and shows partial localization at heterotypic adhesion sites (arrowhead) between an MCF-7 cell and a fibroblast (asterisk). B) N-cadherin is localized at homotypic adhesion sites (arrow) between fibroblasts (asterisks), and shows partial localization at heterotypic adhesion sites (arrowhead). C) P-cadherin is localized only at adhesion sites (arrows) between MCF-7 cells (asterisks), and not at heterotypic adhesion sites (arrowhead). D) OB-cadherin localizes at adhesion sites (arrow) between fibroblasts (asterisk), but not at heterotypic adhesion sites (arrowhead). Scale = 10 µm.
(TIF)

Table S1 Cadherin Expression. Expression, as determined by RT-PCR, of various cadherin family members in MCF-7 cell cultures, NBF cultures, and co-cultures grown for one hour (1 h) or three days (3 d). The first column shows the most common names of the cadherins tested. + indicates expression, and − indicates that expression was not detected by PCR.
(DOC)
