# Supplementary figures and images for: Cadherin-23 Mediates Heterotypic Cell-Cell Adhesion between Breast Cancer Epithelial Cells and Fibroblasts
Source: PLoS One. 2012 Mar 7;7(3):e33289. doi: 10.1371/journal.pone.0033289 (PMC3296689; doi:10.1371/journal.pone.0033289)

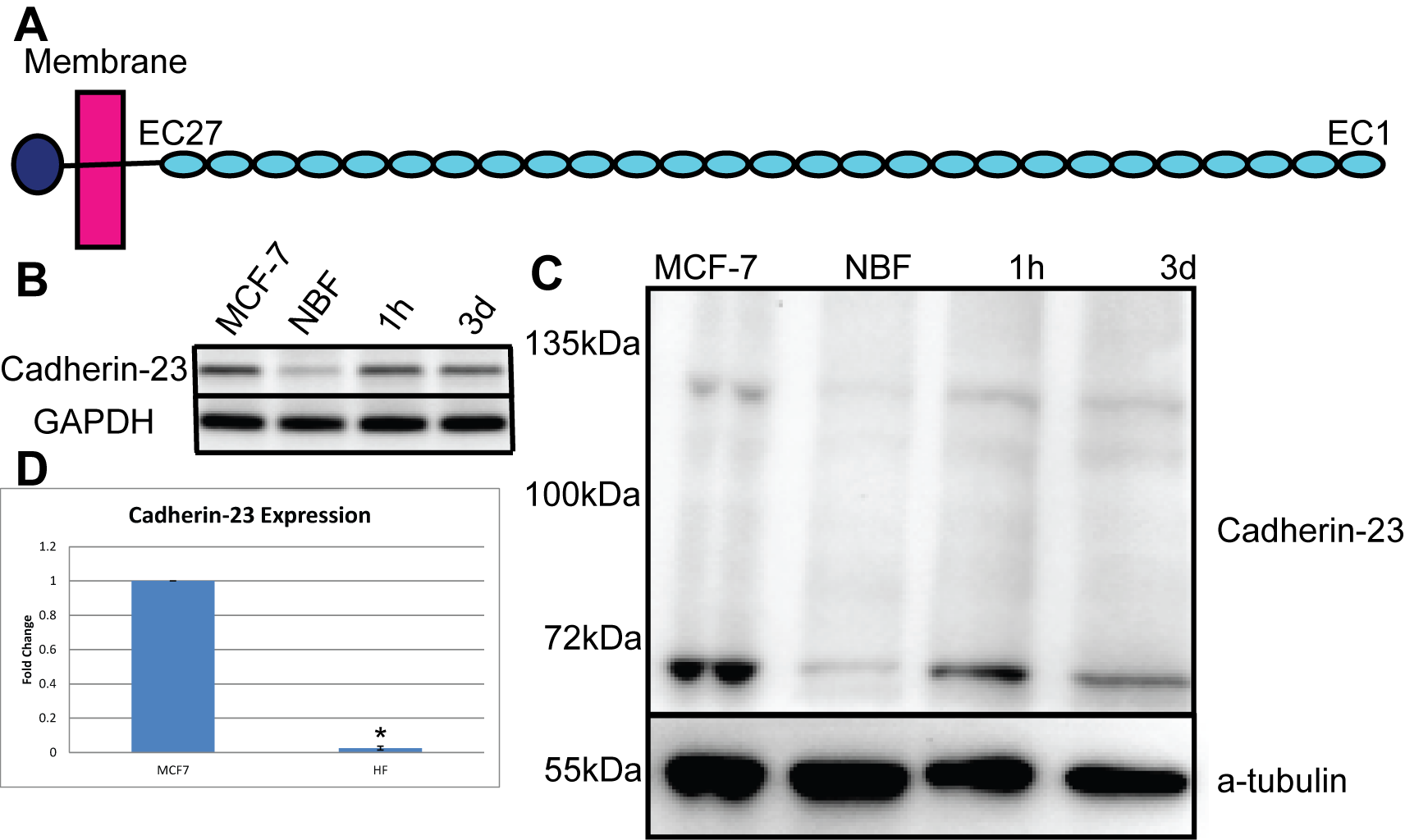

Supplement: Figure S1 — Cadherin-23 is expressed in both MCF-7 cells and NBF cells. A) Domain structure of cadherin-23. B) PCR comparing the expression of cadherin-23 and GAPDH (as a control) in MCF-7 cells, NBF cells and cells co-cultured for 1 hour or 3 days. C) Immunoblot showing protein expression of cadherin-23 and α-tubulin (as a control) in MCF-7 cells, NBF cells and cells co-cultured for 1 hour or 3 days. D) qPCR comparing the expression of cadherin-23 in MCF-7 and NBF mono-cultures. Fold change of cadherin-23 for NBF mono-cultures was compared to that of MCF-7 mono-cultures, which was set as 1. Error bars represent standard error from three independent experiments. Asterisk denotes statistically significant difference (student's t-test, p<0.01). (TIF) [file pone.0033289.s001.tif]

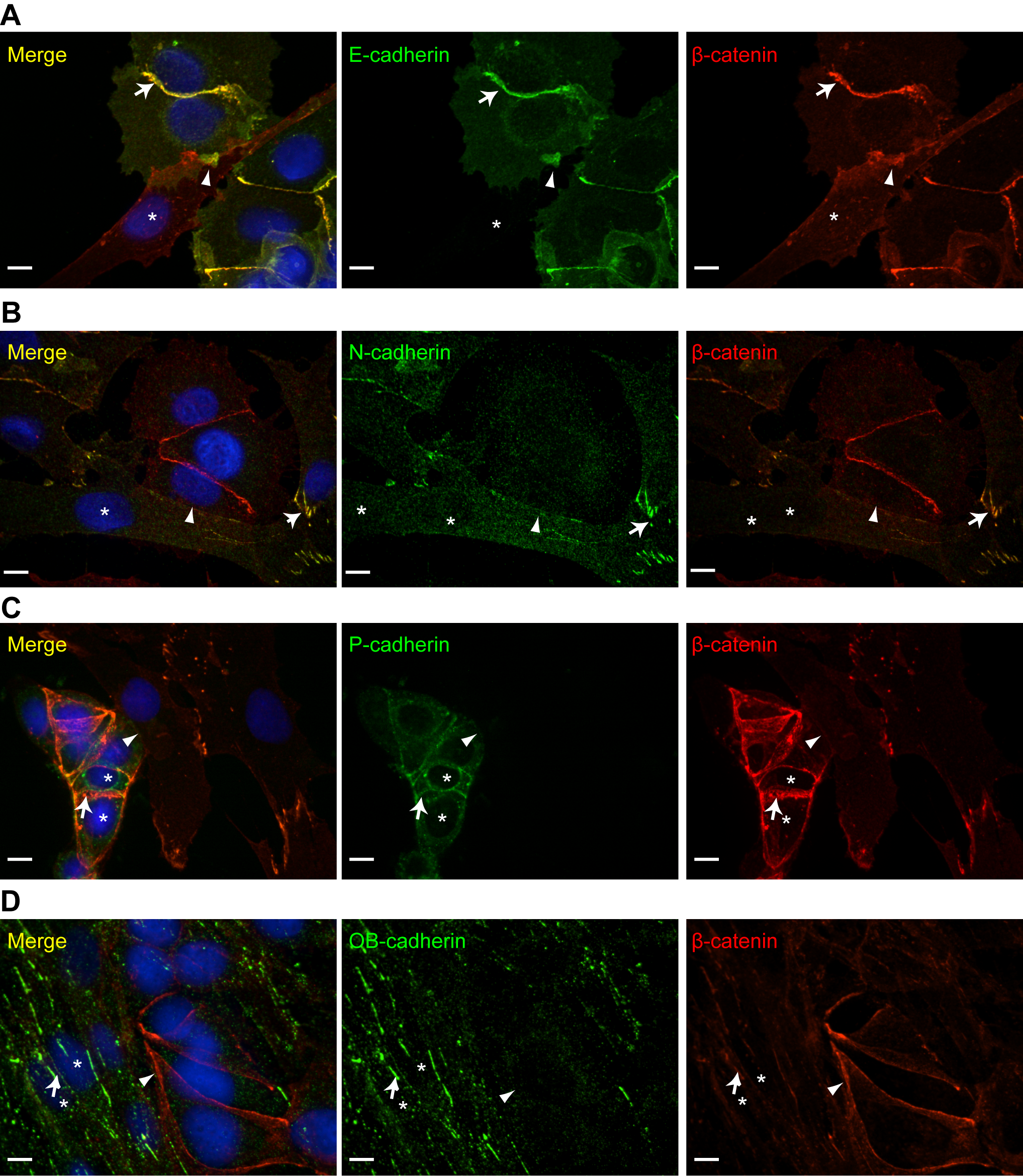

Supplement: Figure S2 — Cadherin localization at homotypic and heterotypic cell-cell contacts. Three-day co-cultures labeled with antibodies to the noted cadherins (green) and β-catenin (red). Nuclei were labeled with DAPI (blue). A) E-cadherin is localized at homotypic adhesion sites (arrow) between MCF-7 cells, and shows partial localization at heterotypic adhesion sites (arrowhead) between an MCF-7 cell and a fibroblast (asterisk). B) N-cadherin is localized at homotypic adhesion sites (arrow) between fibroblasts (asterisks), and shows partial localization at heterotypic adhesion sites (arrowhead). C) P-cadherin is localized only at adhesion sites (arrows) between MCF-7 cells (asterisks), and not at heterotypic adhesion sites (arrowhead). D) OB-cadherin localizes at adhesion sites (arrow) between fibroblasts (asterisk), but not at heterotypic adhesion sites (arrowhead). Scale = 10 µm. (TIF) [file pone.0033289.s002.tif]
