# Supplementary material for: Cadherin-23 Mediates Heterotypic Cell-Cell Adhesion between Breast Cancer Epithelial Cells and Fibroblasts
Source: PLoS One. 2012 Mar 7;7(3):e33289. doi: 10.1371/journal.pone.0033289 (PMC3296689; doi:10.1371/journal.pone.0033289)
Supplement: Table S1 — Cadherin Expression. Expression, as determined by RT-PCR, of various cadherin family members in MCF-7 cell cultures, NBF cultures, and co-cultures grown for one hour (1 h) or three days (3 d). The first column shows the most common names of the cadherins tested. + indicates expression, and − indicates that expression was not detected by PCR. (DOC) [file pone.0033289.s003.doc]

| **Cadherin Common Names** | **MCF7** | **NBF** | **1h** | **3d** | **Common Expression** |
| --- | --- | --- | --- | --- | --- |
| E-cadherin (1) | + | - | + | + | Epithelial |
| N-cadherin (2) | - | + | + | + | Neuronal, Fibroblast, Endothelium |
| P-cadherin (3) | + | - | + | + | Basal Cells of Epidermis |
| R-cadherin (4) | - | - | - | - | Retinal |
| VE-cadherin (5) | - | - | - | - | Vascular Epithelium |
| K-cadherin (6) | - | - | - | - | Fetal Kidney |
| Cadherin 7 | - | - | - | - | Brain |
| Cadherin 8 | - | - | - | - | Brain |
| T1-cadherin (9) | - | - | - | - | Brain, Retina |
| T2-cadherin (10) | - | - | - | - | Brain |
| OB-cadherin (11) | - | + | + | + | Osteoblast Fibroblast |
| N-cadherin 2 (12, Br) | - | - | - | - | Brain |
| H-cadherin (13) | - | - | - | - | Heart, Muscle, Liver, Skin, Neural |
| M-cadherin (15) | - | - | - | - | Myotubule |
| KSP-cadherin (16) | - | - | - | - | Kidney |
| LI-cadherin (17) | - | - | - | - | Liver, Intestine |
| Cadherin 18 | - | - | - | - | CNS |
| Cadherin 19 | - | - | - | - | Neural Crest |
| Cadherin 20 | - | - | - | - | Placenta, Brain |
| Cadherin 22 | - | - | - | - | Brain |
| Cadherin 23 | + | + | + | + | Neurosensory Epithelium |
| Cadherin 24 | - | - | - | - | Brain, Heart, Liver, Lung, Kidney, Pancreas, Placenta, Skeletal Muscle |
